# Uterotubal junction prevents chlamydial ascension via innate immunity

**DOI:** 10.1371/journal.pone.0183189

**Published:** 2017-08-10

**Authors:** Yuyang Zhang, Lili Shao, Xiaodong Li, Guangming Zhong

**Affiliations:** 1 Department of Obstetrics and Gynecology, the First Affiliated Hospital of Wenzhou Medical University, Wenzhou, Zhejiang Province, PR China; 2 Department of Microbiology and Immunology, University of Texas Health Science Center at San Antonio, Texas, United States of America; Midwestern University, UNITED STATES

## Abstract

Ascension to the oviduct is necessary for *Chlamydia* to induce tubal infertility. Using the *Chlamydia muridarum* induction of hydrosalpinx mouse model, we have demonstrated a significant role of the uterotubal junction in preventing chlamydial ascending infection. First, delivery of *C*. *muridarum* to either side of the uterotubal junction resulted in significant reduction in live organisms from the tissues on the opposite sides. However, the recovery yields remained similar among different sections of the uterine horn. These observations suggest that the uterotubal junction may function as a barrier between the uterine horn and oviduct. Second, deficiency in innate immunity signaling pathways mediated by either MyD88 or STING significantly compromised the uterotubal junction barrier function, permitting *C*. *muridarum* to spread freely between uterine horn and oviduct. Finally, transcervical inoculation of *C*. *muridarum* led to significantly higher incidence of bilateral hydrosalpinges in the STING-deficient mice while the same inoculation mainly induced unilateral hydrosalpinx in the wild type mice, suggesting that the STING pathway-dependent uterotubal junction plays a significant role in preventing tubal pathology. Thus, we have demonstrated for the first time that the uterotubal junction is a functional barrier for preventing tubal infection by a sexually transmitted agent, providing the first *in vivo* evidence for detecting chlamydial infection by the STING pathway.

## Introduction

*Chlamydia trachomatis* is a leading infectious cause of tubal infertility in women [1–3]. However, the mechanisms by which *C*. *trachomatis* causes tubal infertility remain unknown. *Chlamydia muridarum* infection in the mouse genital tract, which can cause hydrosalpinx and infertility [4–6], has been a useful model for investigating the chlamydial pathogenic mechanisms. The model has allowed the identification of both chlamydial [7–11] and host [12–20] factors involved in chlamydial induction of upper genital tract pathology.

Intravaginal inoculation with *C*. *muridarum* leads to chlamydial spread from the lower genital tract, through the cervical barrier, into the endometrial cavity. Compared to intravaginal inoculation, transcervical inoculation of *C*. *muridarum* directly into uterine or endometrial cavity (thus, bypassing the cervical barrier) resulted in significantly higher incidence and more severe hydrosalpinges in many strains of mice [6, 21, 22], indicating an important role of the cervical barrier in protecting the upper genital tract. However, not all transcervically inoculated mice developed more severe hydrosalpinx. This is because the endometrial *C*. *muridarum* organisms have to further ascend through the uterotubal junction into the oviduct in order to induce hydrosalpinx and tubal infertility. It is unclear, at this moment, whether the uterotubal junction, connecting the endometrial cavity of the uterine horn at the distal end with the oviduct at the proximal tubal opening, can prevent the endometrial chlamydial organisms from ascending to the oviduct. The uterotubal junction covers the genital tract region, in which the endometrial epithelium changes over to the ciliated tubal epithelium and is known to serve as a selective gate for passage of individual spermatozoa with normal morphology [23], leading to predominantly normal sperm passing towards the ovum [24]. Furthermore, the uterotubal junction may also play a significant role in host defense since *E*. *coli* bacteria experimentally delivered into the endometrial cavity were restricted from ascending to the oviduct or vice versa [25]. Thus, we hypothesize that the uterotubal junction may also limit the ascension of the sexually transmitted *Chlamydia* from the endometrial cavity to the oviduct.

Chlamydial infection is known to activate innate immunity signaling pathways, including those mediated by MyD88 [14, 26] and STING [27, 28] and to induce inflammatory cytokines [12, 29] and both cellular [30–33] and soluble effectors [34, 35]. Since *Chlamydia* is capable of rapid ascending infection [36], the early-induced host responses may play significant roles in preventing chlamydial ascension. For example, MyD88 deficiency significantly increased mouse susceptibility to *C*. *muridarum* ascending infection [14], suggesting that the MyD88-regulated mucosal effectors at either the cervical or uterotubal junction barriers may contribute to the prevention of chlamydial ascension. Furthermore, Chlamydia is known to induce type I interferon via the activation of the STING pathway in cell cultures [27, 28]. However, the role of STING-mediated signaling pathway in chlamydial infection has not been evaluated in animal models.

In the current study, we evaluated the role of uterotubal junction in preventing *C*. *muridarum* from ascending to the oviduct. We found that either intrabursal or intrauterine delivery of *C*. *muridarum* led to significant reduction in the number of organisms in the opposite sides of the uterotubal junction, indicating a barrier effect between the mouse uterine horn and oviduct. Furthermore, deficiency in innate immunity signaling pathways mediated by either MyD88 or STING significantly compromised the uterotubal junction barrier effect. Finally, *C*. *muridarum* induced significantly higher incidence of bilateral hydrosalpinges in the STING-deficient mice compared to the wild type mice. These observations have demonstrated that the STING pathway-dependent uterotubal junction function represents a significant host defense mechanism for reducing tubal pathology.

## Materials and methods

### *Chlamydia muridarum* and mouse infection

*Chlamydia muridarum* strain Nigg organisms (initially acquired from Dr. Robert C. Brunham’s lab at the University of Manitoba in 1999) were propagated and purified in HeLa cells (human cervical carcinoma epithelial cells, ATCC cat# CCL2) as described previously [31, 37]. After comparing its full genome sequence (Genbank accession# CP009760.1) with that of the reference *C*. *muridarum* [38], we re-designated this strain as Nigg3. A plaque-cloned strain (designated as G13.32.1) from Nigg3 was used in the current study [8, 9]. The *C*. *muridarum* organisms were purified as elementary bodies (EBs) and stored in aliquots at -80°C until use. Six to seven week-old female C57BL/6J (stock number 000664), Balb/c (000651), CBA/J (000656), MyD88-deficient (009088) and Goldenticket (with a missense mutation in *Sting* gene, 017537, thus, also called STING-deficient, ref; [39]; all from Jackson Laboratories, Inc., Bar Harbor, Maine) mice were inoculated with *C*. *muridarum* at 2 X 10^5^ inclusion-forming units (IFUs) via different routes as indicated in individual experiments. After inoculation, mice were sacrificed on different days as indicated in individual experiments for monitoring live chlamydial organism recovery from different sections of the genital tract. The intrabursal infection was carried out as described previously [22]. Briefly, mice were anesthetized and laid with the dorsal side up on a sterile gauze pad with the mouse head facing away. A small incision was made at the dorsomedial position and directly above the ovarian fat pad. After the ovarian fat pad was gently pulled out, the ovary was positioned to allow for insertion of a needle (30GA, Removable needles, Hamilton, #7803–07) into the oviduct tubule. When the needle was inserted into the proper position, it was visible under the bursa. The plunger of the syringe (Hamilton, #7654–01) was gently pushed to inject the 10μl of inoculum, after which the needle was quickly removed and the puncture site was gently sealed. The bursa should be slightly distended if the injection is successful. Finally, the reproductive tract and fat pad were gently put back into the peritoneal cavity and the body wall was closed and sutured (Reli sutures, SK683, Busan, South Korea). After recovery, the mice were returned to cages for normal care. For intrauterine horn inoculation, we used the same procedure as described above, except that the inoculum was injected to the distal end of the uterine horn. All inoculations were carried out at the right side of the mice for both consistency and convenient monitoring of live organism spreading. For transcervical inoculation, a Non-Surgical Embryo Transfer Device (NSET, cat# 60010, ParaTechs Corp., Lexington, KY) was used, and the manufacturer’s instruction (http://www.paratechs.com/nset/) was followed as described previously [22]. The only modification in this experiment was that the embryo transfer device was inserted slightly deeper towards one side so that most inoculum was delivered to one side of the mouse uterus. For survival surgeries (intrabursal or intrauterine injection), 3% Isoflurane was used to anesthetize mice via inhalation. We made sure that mice completely loss consciousness before operation by using a needle to pinch the toe. If no response, surgery might start. Each surgery only lasted about 3 to 4 min. During the surgery, 1% isoflurane inhalation was maintained. After completing surgery and sewing, mice were kept under 1% isoflurane for a couple of minutes, then, placed the recovered animal back to the cage. Mice were provided a safe heat source to avoid hypothermia and to speed up recovery. The breathing rate was monitored. If mice breathe with ease and the return of muscle tone and the ability to voluntarily move were noted, the mice were in the process of recovery. To relieve post surgery discomfort, the following post-operative analgesia was given to all animals: 0.1mg/ml of ibuprofen in the water for 3 days.

All animal experiments were carried out in accordance with the recommendations in the Guide for the Care and Use of Laboratory Animals of the National Institutes of Health. All mice were sacrificed at the conclusion of the corresponding experiments using overdose isoflurane followed by cervical dislocation. The protocol was approved by the Committee on the Ethics of Laboratory Animal Experiments of the University of Texas Health Science Center at San Antonio.

### Titrating live chlamydial organisms recovered from mouse genital tract tissues

For monitoring live organism shedding, mice were sacrificed for harvesting the genital tract tissues at different time points, including 12h, 24h, 48h, 72h, 96h or 14 days, post inoculation. The genital tract tissues were harvested in the following sections, including right oviduct/ovary, right uterine horn (distal, middle and proximal were sometimes harvested separately), vagina/cervix, left uterine horn (proximal, middle and distal) and left oviduct/ovary. To quantitate live chlamydial organisms, each tissue section was soaked in 0.5ml of SPG for homogenization using a 2mL tissue grinder (cat# K885300-0002, Fisher scientific, Pittsburg, PA) or an automatic homogenizer [Omni Tissue Homogenizer, TH115, Kennesaw, GA]. The homogenates were briefly sonicated and spun at 3000rpm for 5min to pellet remaining debris. The supernatants after centrifugation were titrated for live *C*. *muridarum* organisms on HeLa cells as described previously [7]. The infected cultures were processed for immunofluorescence assessment as described previously [10, 21]. Inclusions were counted in five random fields per coverslip under a fluorescence microscope. For coverslips with less than one IFU per field, entire coverslips were counted. Coverslips showing obvious cytotoxicity of HeLa cells were excluded. The total number of IFUs per tissue was calculated based on the mean IFUs per view, the ratio of the view area to that of the well, dilution factor, and inoculation volumes. Where possible, a mean IFU/tissue was derived from the serially diluted samples for any given tissue. The total number of IFUs/tissue was converted into log_10_ and used to calculate the mean and standard deviation across mice of the same group at each time point. The results were expressed as log IFUs per organ or tissue segment.

### Evaluating genital tract gross pathology and inflammatory infiltration

Seventy days after infection, mice were euthanized for harvesting genital tracts. Gross pathology of hydrosalpinx was observed and documented by high-resolution digital photography. Hydrosalpinx was scored according to an ordinal scale where 0 indicates no hydrosalpinx, 1 indicates hydrosalpinx that is only observable under magnification, 2 indicates visible hydrosalpinx smaller than the size of the ovary, 3 indicates hydrosalpinx roughly equal to the size of the ovary, and 4 indicates hydrosalpinx larger than the ovary. Bilateral hydrosalpinx severity was calculated for each mouse as the summed scores of the left and right oviducts. Hydrosalpinx incidence was calculated as the number of mice with a bilateral score of 1 or higher divided by the total number of mice in the group. Chronic inflammation or infiltration of mononuclear cells into the genital tract tissues was evaluated histologically. Following gross pathology assessment, whole genital tracts were fixed in 10% neutral formalin, stored in 70% ethanol in water, embedded in paraffin, and serially sectioned longitudinally across the plane of the whole genital tract at 5μm intervals on an AccuCut SRM 200 microtome (Sakura, Torrance, CA). Three nearly equidistant sections at roughly 5μm, 30μm, and 55μm into the lateral surface of the oviduct were subjected to hematoxylin and eosin (H&E) staining, sealed on glass tissue slides, and observed under a microscope for infiltration of mononuclear inflammatory cells into tissue surrounding the oviduct. These infiltrates were scored on an ordinal scale where 0 represents no cellular foci, 1 indicates a single focus, 2 indicates two to four loci, 3 indicates more than four foci, and 4 indicates confluent infiltration. The median of the three scores served as a single value for each oviduct unilateral inflammation score, and both unilateral scores for each mouse were combined to form a bilateral score.

### Statistics analyses

The incidence rates were analyzed using Fisher’s exact test while the number of live organisms (IFUs) and pathology scores were analyzed using the Wilcoxon rank sum test for comparing any two groups.

## Results

### Uterotubal junction is a functional barrier for preventing spread of *C*. *muridarum* organisms across the mouse genital tract

**Fig 1:**
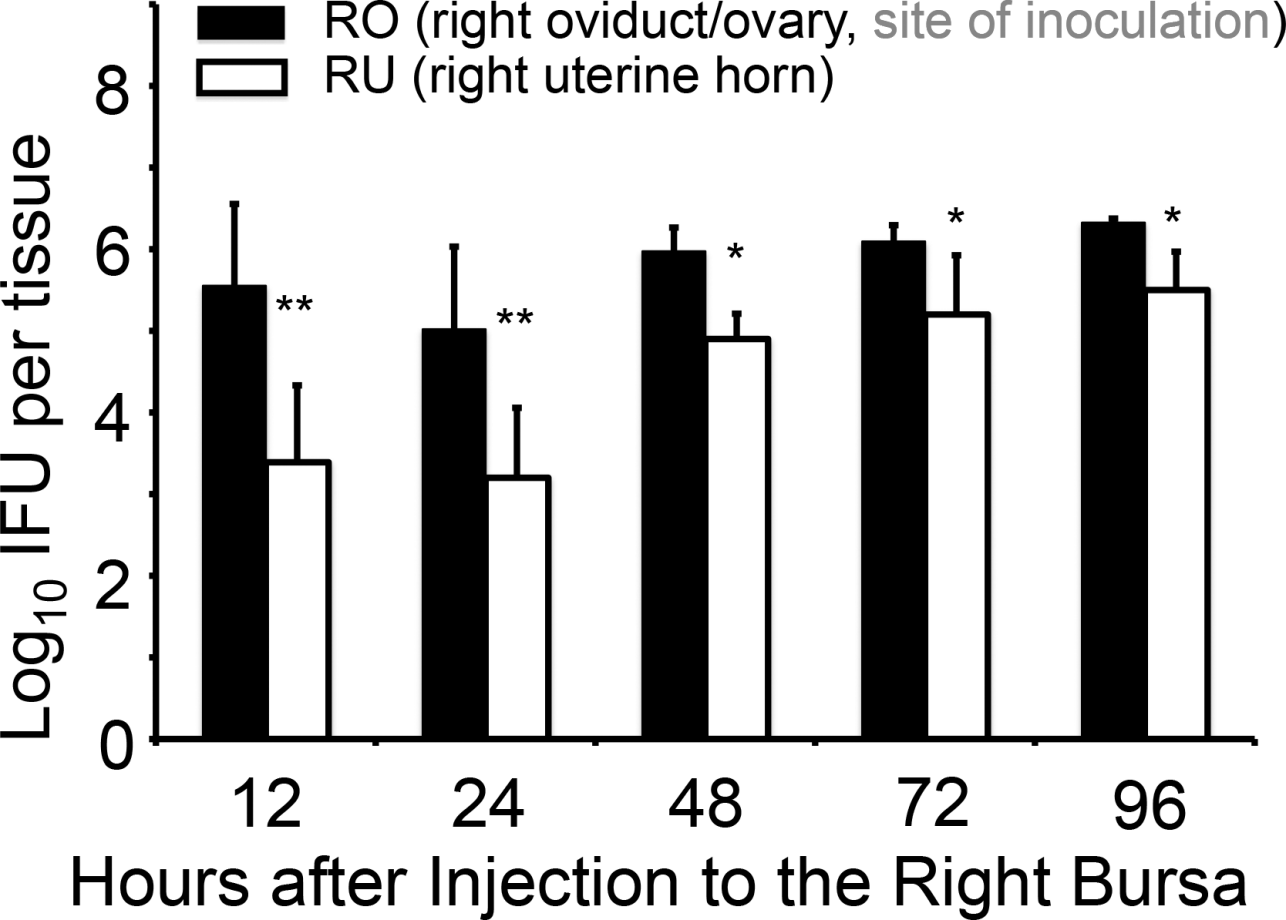
Reduced spreading of *Chlamydia muridarum* from the oviduct/ovary into uterine horn after intrabursal injection. The wild type *C*. *muridarum* organisms were intrabursally inoculated into the right oviduct/ovary of female C57BL/6J mice with 2 x 10^5^ inclusion forming units (IFUs) per mouse. At various time points post inoculation as indicated along the X-axis, groups of mice (n = 4 to 5) were sacrificed for harvesting the right oviduct/ovary (RO, solid bar) and right uterine horn (RU, open) respectively. The tissue samples were homogenized for titrating *C*. *muridarum* live organisms, and the titers were expressed as Log10 IFUs displayed along the Y-axis. Please note that titers of live organisms recovered from RO that received direct injection were always significantly higher than those from RU that is on the opposite side of the uterotubal junction (**p<0.01, *p<0.05, Wilcoxon), suggesting a functional barrier between the oviduct and uterine horn. The lower recovery during the first 24h is consistent with the concept that the inoculum needs time to replicate and to differentiate the replicating but non-infectious reticulate bodies into the infectious elementary bodies.

To determine whether mouse uterotubal junction can serve as a barrier for limiting the lumenal spreading of *C*. *muridarum*, we inoculated live *C*. *muridarum* organisms into the right bursa of female C57BL/6J mice and monitored live organism distribution in both the right oviduct/ovary and right uterine horn tissues at different time points after the inoculation (Fig 1). We found that the inoculum was almost fully recovered from the injection site, the right oviduct/ovary tissue, at 12 hours after inoculation. However, 100 fold less organisms were recovered in the adjacent uterine horn tissue. By 24 hours after the inoculation, the recovered live organisms decreased from both the right oviduct/ovary and uterine horn tissues, which may reflect either chlamydial differentiation into non-infectious reticulate bodies or mucosal killing of chlamydial organisms. However, by 48 hours, the chlamydial organisms increased in both tissue samples, but the yield from the uterine horn tissue was still 10 fold lower than that from the oviduct/ovary tissue. This trend continued to 96 hours after inoculation. The observation that live organisms recovered from the oviduct/ovary tissue samples were always significantly higher than those recovered from the uterine horn tissues suggests a barrier function between the oviduct/ovary and uterine horn.

**Fig 2:**
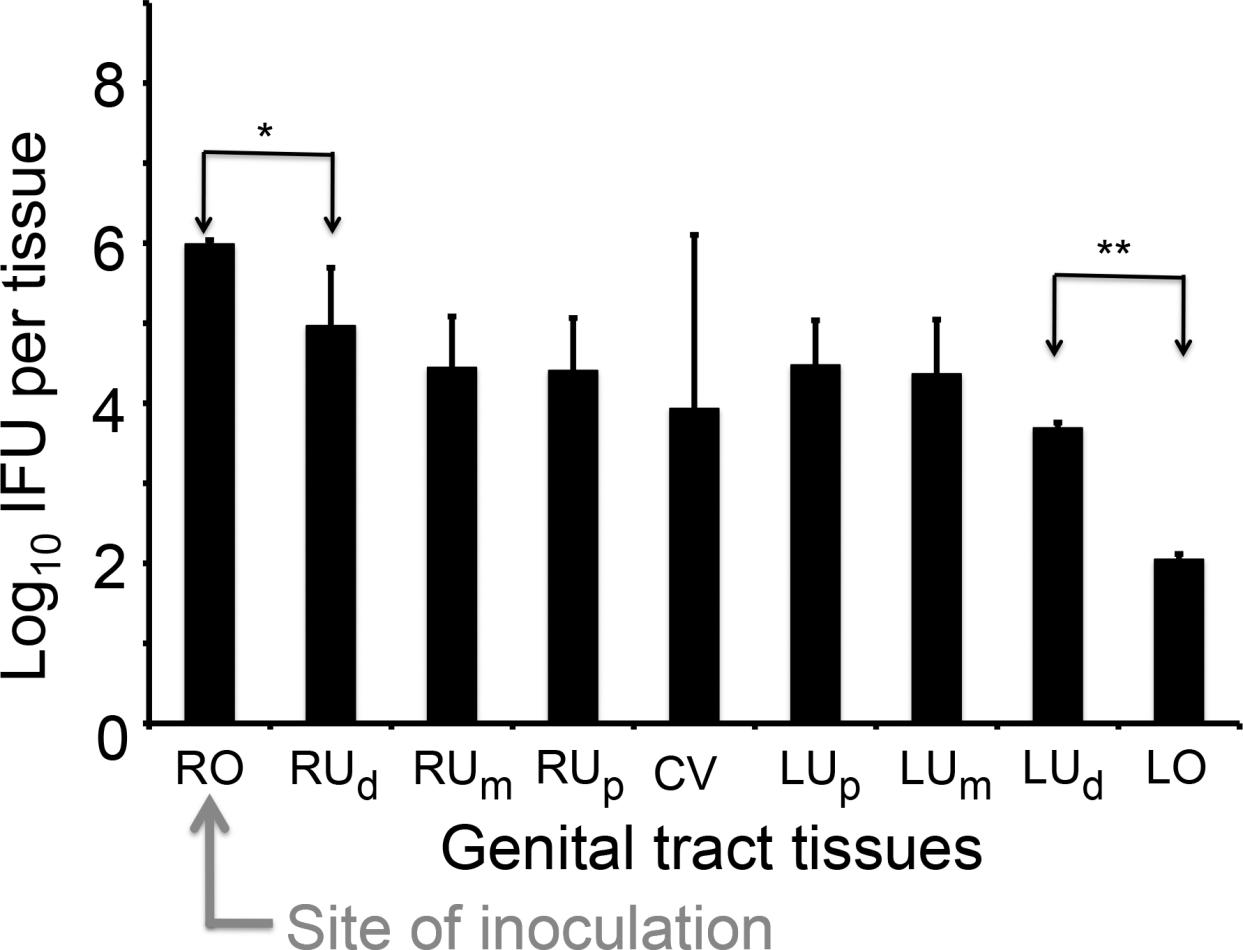
Comparing *C*. *muridarum* recoveries from 9 different sections of the mouse genital tract following inoculation into the right bursa. A group of 5 C57BL/6J female mice were each inoculated with 2 x 10^5^ IFUs at the right bursa (as indicated with a red arrow) and 3 days after the inoculation, segments of the entire genital tract tissues were harvested as shown along with the X-axis, from the right oviduct/ovary (RO), right uterine horn distal region (RU_d_), middle (RU_m_) or the proximal region (RU_p_), cervico-vagina (CV), left side uterine horn proximal region (RU_p_), middle (LU_m_) and distal (RU_d_) to the left oviduct/ovary (LO). The tissues were homogenized for titrating infectious organisms as displayed in Log10 IFUs along the Y-Axis. Please note that significant differences in log10 IFUs were observed only between RO and RU_d_ (*p<0.05, Wilcoxon) and between LU_d_ and LO (**p<0.01, Wilcoxon) but not other adjacent tissue sections.

To test whether such a barrier function is only restricted to the uterotubal junction between the oviduct and uterine horn, we repeated the above experiment by comparing live organism recovery from 9 different sections of the genital tract at 72 hours after inoculation (Fig 2). We found that besides the significant difference in live organism recovery between the inoculated tissue, the right oviduct/ovary, and the right uterine horn distal region, the recovery from the left ovary/oviduct was significantly lower than that of the left uterine horn distal region. The difference was greater than 100 fold. However, the recovery yields among the remaining genital tract tissue sections were less variable. For example, the number of live organisms recovered from the cervicovaginal (CV) tissue and the tissues from the left or right uterine segments differed from each other by less than 10 folds. These observations have further validated the barrier function of the uterotubal junction between the uterine horn and oviduct. It is worth noting that the uterotubal junction barrier restricted *C*. *muridarum* spreading from both directions, either descending from the right oviduct to the right uterine horn or ascending from the left uterine horn to the left oviduct.

**Fig 3:**
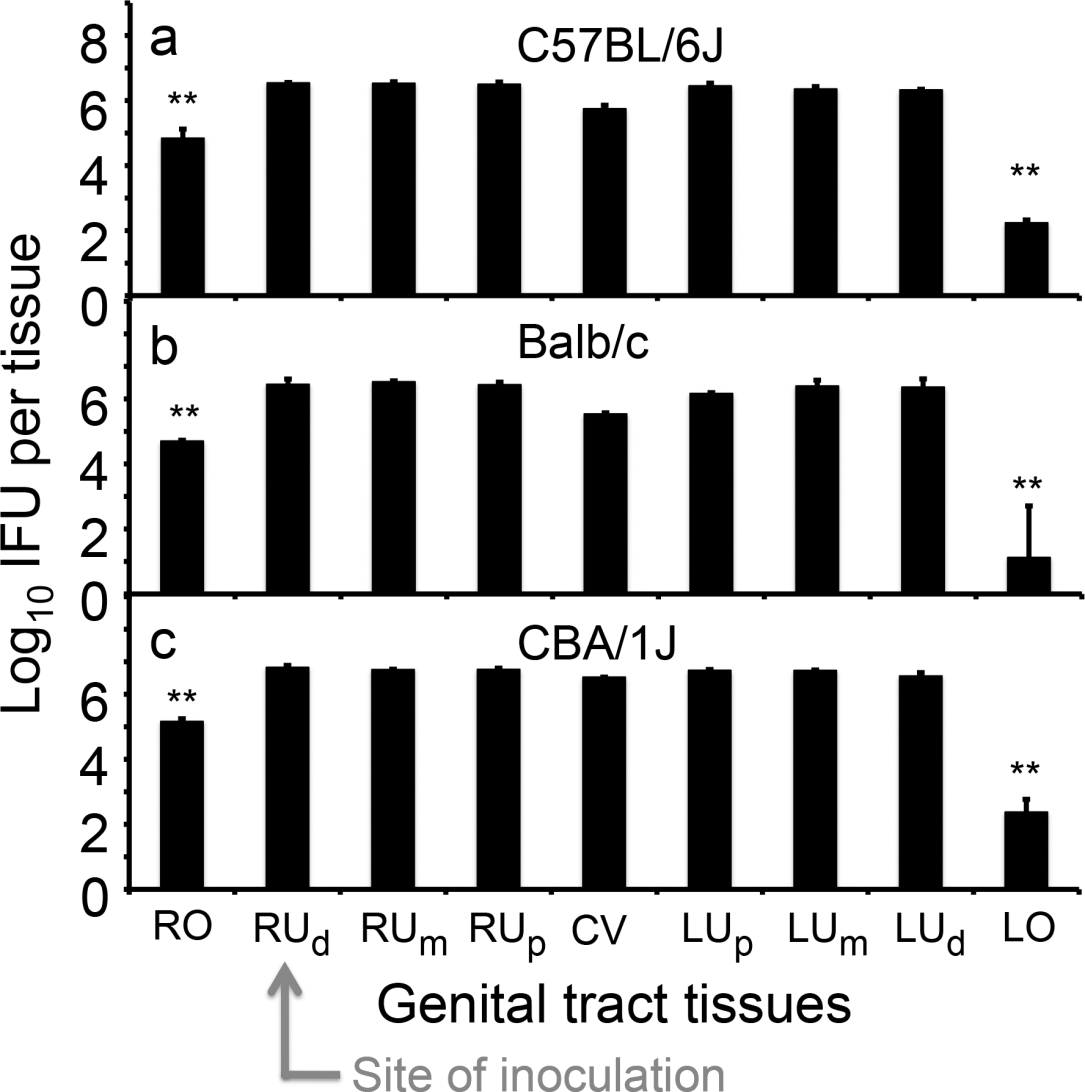
Comparing *C*. *muridarum* distribution in genital tract among three different mouse strains. Groups of C57BL/6J (panel a, n = 5), Balb/c (b, n = 5) and CBA/1J (c, n = 5) female mice were each inoculated with 2 x 10^5^ IFUs at the right uterine horn distal region (RU_d_, as indicated with a red arrow) and 3 days after the inoculation, segments of the genital tract tissues were harvested as listed along the X-axis, from RO, RU_d_, RU_m,_ RU_p_, CV, LU_p_, LU_m_, RU_d_ to LO; tissue designations are indicated in Fig 2 legend The tissues were homogenized for titrating infectious organisms as displayed in Log10 IFUs along the Y-Axis. Please note that significant differences in log10 IFUs were observed between RO and RU_d_ (**p<0.01, Wilcoxon) and between LU_d_ and LO (**p<0.01, Wilcoxon) in all 3 mouse strains.

Since the above findings were made using C57BL/6J mice, we further tested whether the uterotubal junction could function as a barrier in other mouse strains. We compared the live organism recovery from 9 different sections of the genital tract on day 3 after the inoculation with *C*. *muridarum* to the right uterine horn distal region between C57BL/6J, Balb/c and CBA/1J mice (Fig 3). We found that similar levels of live organisms were recovered from all 6 uterine horn sections and the cervicovaginal tissues in all 3 strains of mice, suggesting that there is no significant barrier effect between these tissues. However, the right oviduct/ovary tissues that were next to the inoculation sites displayed >10 fold lower levels of live organisms than the injected tissues (right uterine horn distal region). More interestingly, although the left uterine horn distal region reached an equilibrium level of live organisms with the rest of the uterine horn tissues, the left oviduct/ovary displayed ~1,000 fold fewer organisms in all 3 strains of mice. Thus, we have demonstrated that the uterotubal junction region can function as a significant barrier for reducing chlamydial spread from the endometrial cavity to the oviduct.

### The uterotubal junction barrier effect is dependent on innate immunity signaling pathways

**Fig 4:**
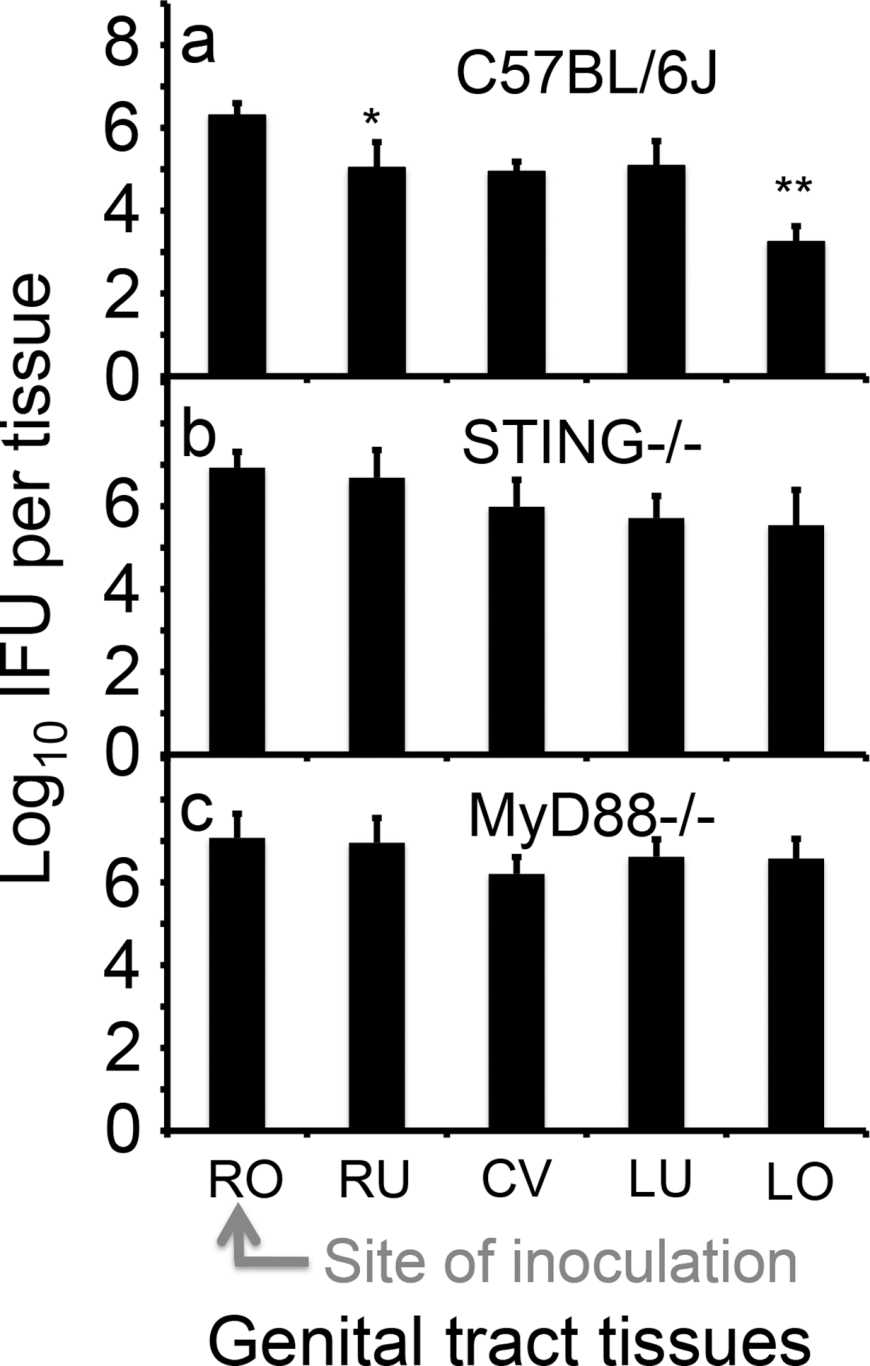
Evaluating the effect of MyD88 or STING pathways on the spread of *C*. *muridarum* in the genital tract following an inoculation to the right bursa. Groups of C57BL/6J (panel a, n = 5), STING deficient (b, STING-/-, n = 5) and MyD88 deficient (c, MyD88-/-, n = 5) female mice were each inoculated with 2 x 10^5^ IFUs at the right bursa (RO, as indicated with a red arrow) and 3 days after the inoculation, segments of the genital tract tissues were harvested as listed along the X-axis, from RO, RU (entire right uterine horn), CV, LU (entire left uterine horn) to LO. The tissues were homogenized for titrating infectious organisms as displayed in Log10 IFUs along the Y-Axis. Please note that C57BL/6J mice displayed significant differences in log10 IFUs between RO and RU (*p<0.05, Wilcoxon) and between LU and LO (**p<0.01, Wilcoxon) respectively but neither STING-deficient nor MyD88-deficient mice were able to maintain the differences.

The uterotubal barrier function was detectable throughout the first 96 hours after inoculation, suggesting that innate immunity may play a significant role. Since chlamydia has been shown to activate both the MyD88 [14, 26] and STING [27, 28] pathways, we tested whether mice deficient in either pathway were compromised in the barrier function (Fig 4). We found that 3 days following inoculation of *C*. *muridarum* into the right bursa, the wild type C57BL/6J mice displayed significant differences in live organism recovery both between the right oviduct/ovary and right uterine tissues and between the left uterine horn and left oviduct/ovary tissues, indicating the barrier function of the uterotubal junction. However, these differences disappeared in mice deficient in either STING or MyD88 (although slightly lower levels of live organisms were detected in the right uterine and left oviduct tissues of the STING-deficient mice), suggesting that a functional innate immunity pathway is required for maintaining the uterotubal junction barrier functions.

**Fig 5:**
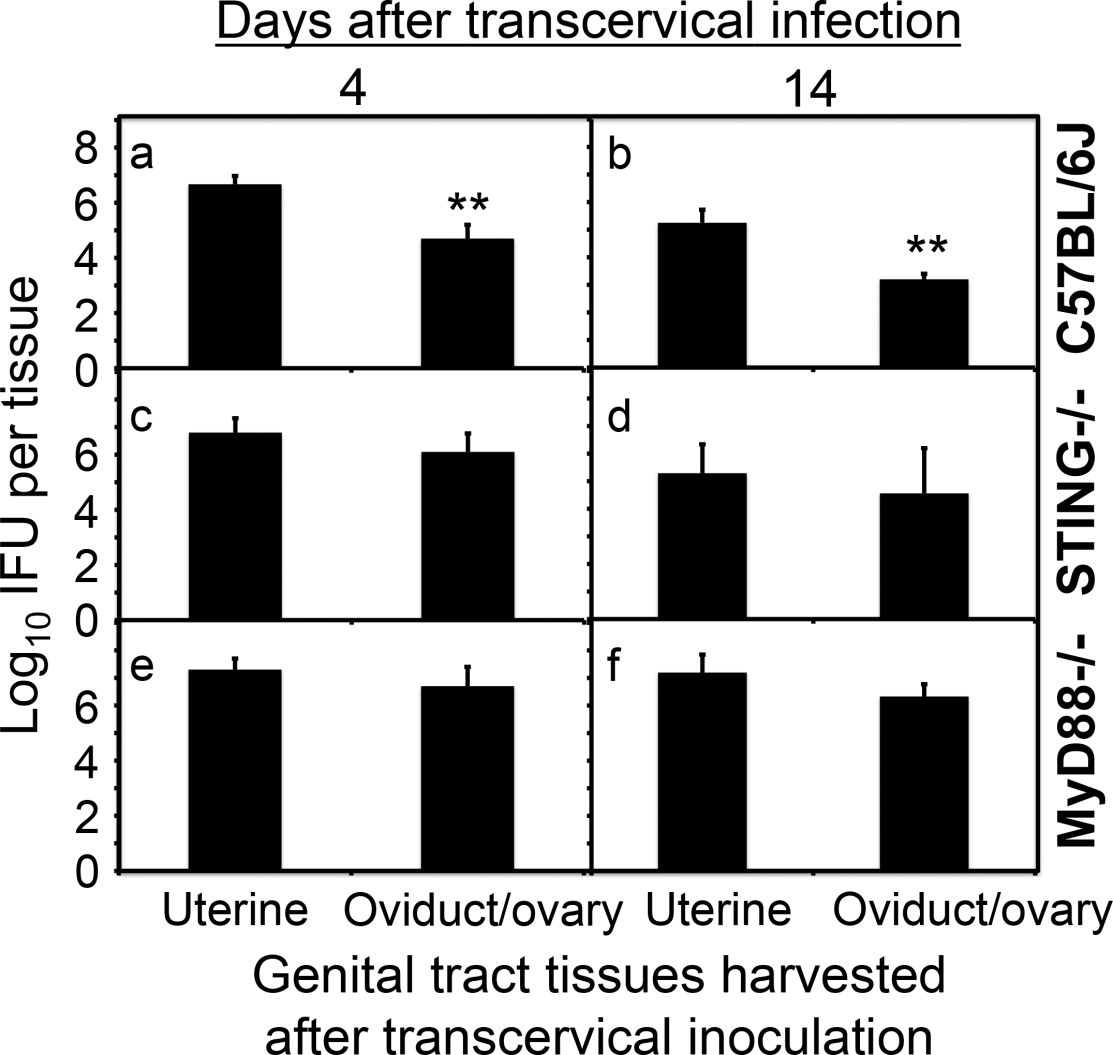
Evaluating the effect of MyD88 or STING pathways on *C*. *muridarum* ascending to the oviduct following a transcervical inoculation. Groups of C57BL/6J (panels a & b, n = 5), STING-deficient (c & d, STNG-/-, n = 5) and MyD88-deficient (e & f, MyD88-/-, n = 5) female mice were each inoculated with 2 x 10^5^ IFUs transcervically and 4 (a, c & e) or 14 (b, d & f) days after the inoculation, the entire uterine horn (both right and left) and oviduct/ovary (both right and left) tissues from each mouse were harvested as listed along the X-axis. The tissues were homogenized for titrating infectious organisms as displayed in Log10 IFUs along the Y-Axis. Please note that C57BL/6J mice displayed significant differences in log10 IFUs between uterine and oviduct/ovary tissues (a & b, **p<0.01, Wilcoxon) but neither STING-deficient nor MyD88-deficient mice exhibited any significant differences.

To test whether the compromised uterotubal junction barrier function has any long-term consequences, we compared the chlamydial ascending infection from the uterine horn to the oviduct/ovary tissues on days 4 and 14 following transcervical inoculation between the wild type C57BL/6J mice and mice deficient in STING or MyD88 (Fig 5). We found that the wild type C57BL/6J mice developed significantly reduced levels of live organisms in the oviduct/ovary tissues on day 4 and the difference was maintained throughout the 14 days after transcervical inoculation, validating the role of uterotubal junction in maintaining sustained barrier function between the uterine horn and oviduct/ovary. The significant reduction in the overall titers of live organisms on day 14 compared to those from day 4 was probably due to the clearance of *C*. *muridarum* by adaptive immunity. Importantly, live organism numbers reached similar levels in both the uterine and oviduct/ovary tissues of mice-deficient in either STING or MyD88. These observations suggest that both STING and MyD88 pathways are required for maintaining a normal barrier function of the uterotubal junction.

### The uterotubal junction protects oviduct from inflammatory pathology induced by *C*. *muridarum*

**Fig 6:**
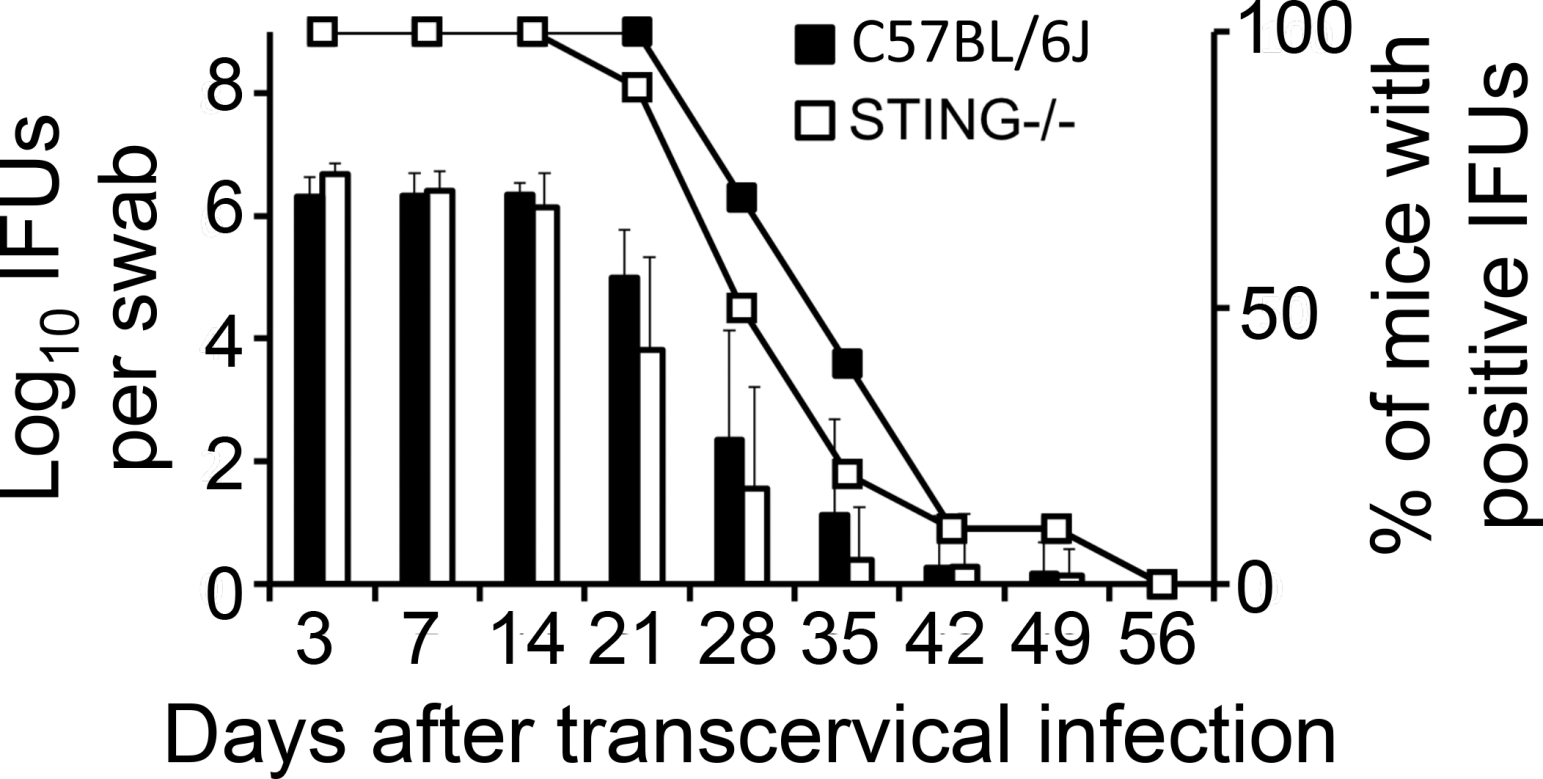
Comparing *C*. *muridarum* recoveries from vaginal swabs of mice with or without deficiency in STING following a transcervical inoculation. Groups of C57BL/6J (solid bar or, solid square, n = 10) and STING-deficient (open bar or open square, n = 10) female mice were each inoculated with 2 x 10^5^ IFUs transcervically and at various times after the inoculation as indicated along the X-axis, vaginal swabs were taken for titrating infectious organisms as displayed in Log10 IFUs (left) or % of mice with positive IFU (right) along the Y-Axis. Please note that there is no significant difference in either log10 IFUs (Wilcoxon) or % of mice with positive IFUs (Fisher’s Exact) between the wild type and STING-deficient mice (data obtained from two independent experiments), indicating that the STING-deficiency did not affect the descending of the *C*. *muridarum* organisms from the endocervcal compartments into the ectocervical and vaginal compartments or the replication of the *C*. *muridarum* organisms in these compartments.

**Fig 7:**
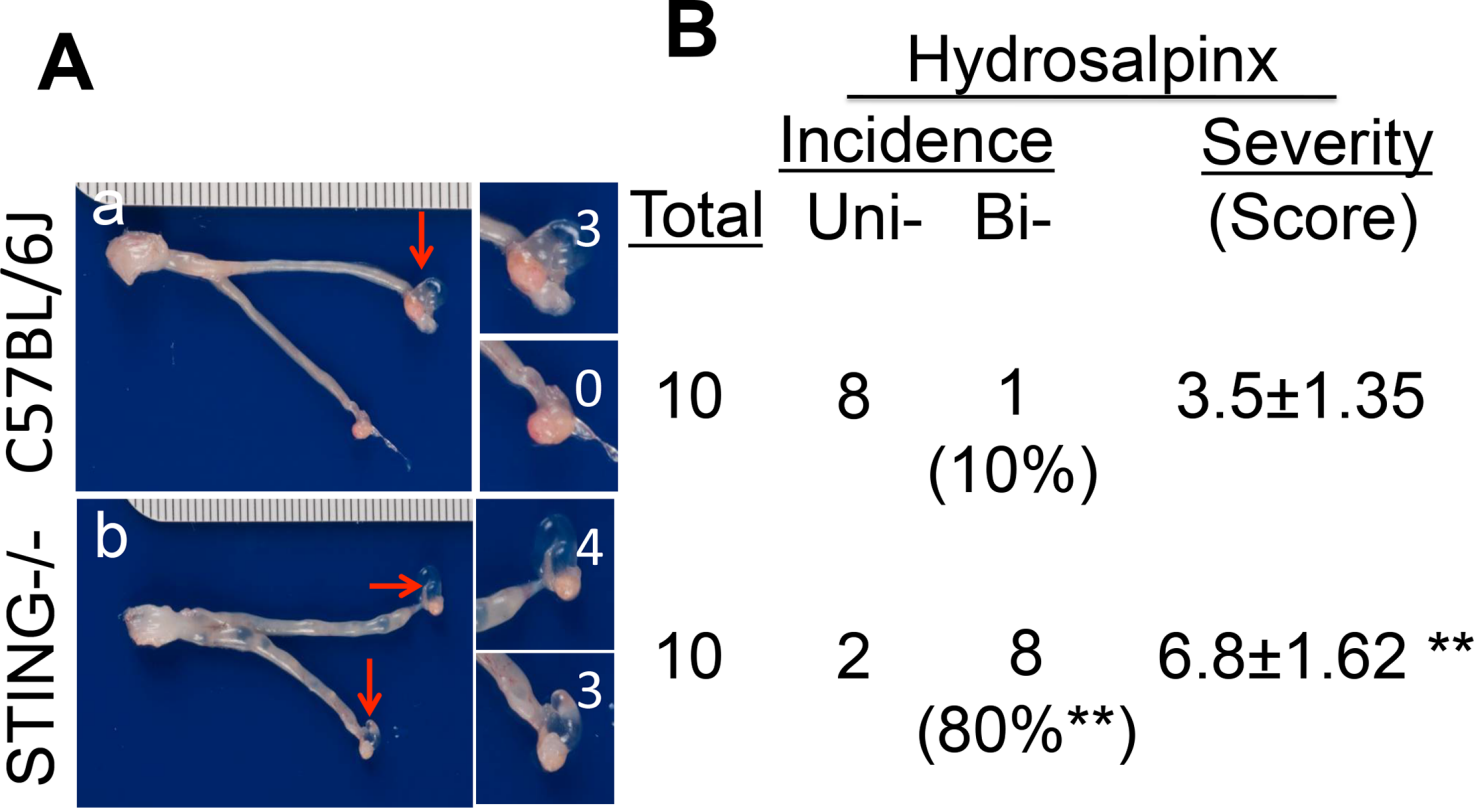
Comparing gross pathology in the oviducts of mice with or without deficiency in STING following a transcervical inoculation. Groups of C57BL/6J (n = 10) and STING-deficient (n = 10) female mice were each inoculated with 2 x 10^5^ IFUs transcervically as described in Fig 6 legend and 70 days after the inoculation, mice were sacrificed for evaluating gross pathology. (A) A representative image is shown from each group (A, panels a & b respectively). Hydrosalpinges were marked with red arrows while the severity of hydrosalpinx was scored based on the semi-quantitative scheme described in the materials and the scores were marked with white numbers. (B) Summary of both the hydrosalpinx incidence and severity. The incidence of mice with unilateral or bilateral hydrosalpinges was counted separately while the severity was scored based on total hydrosalpinges. Please note that only 10% the wild type C57BL/6J mice developed bilateral hydrosalpinx while 80% of the STING-deficient mice did so (**p<0.01, Fisher Exact). The STING-deficient mice also developed more severe hydrosalpinx than the wild type mice (**p<0.01, Wilcoxon).

**Fig 8:**
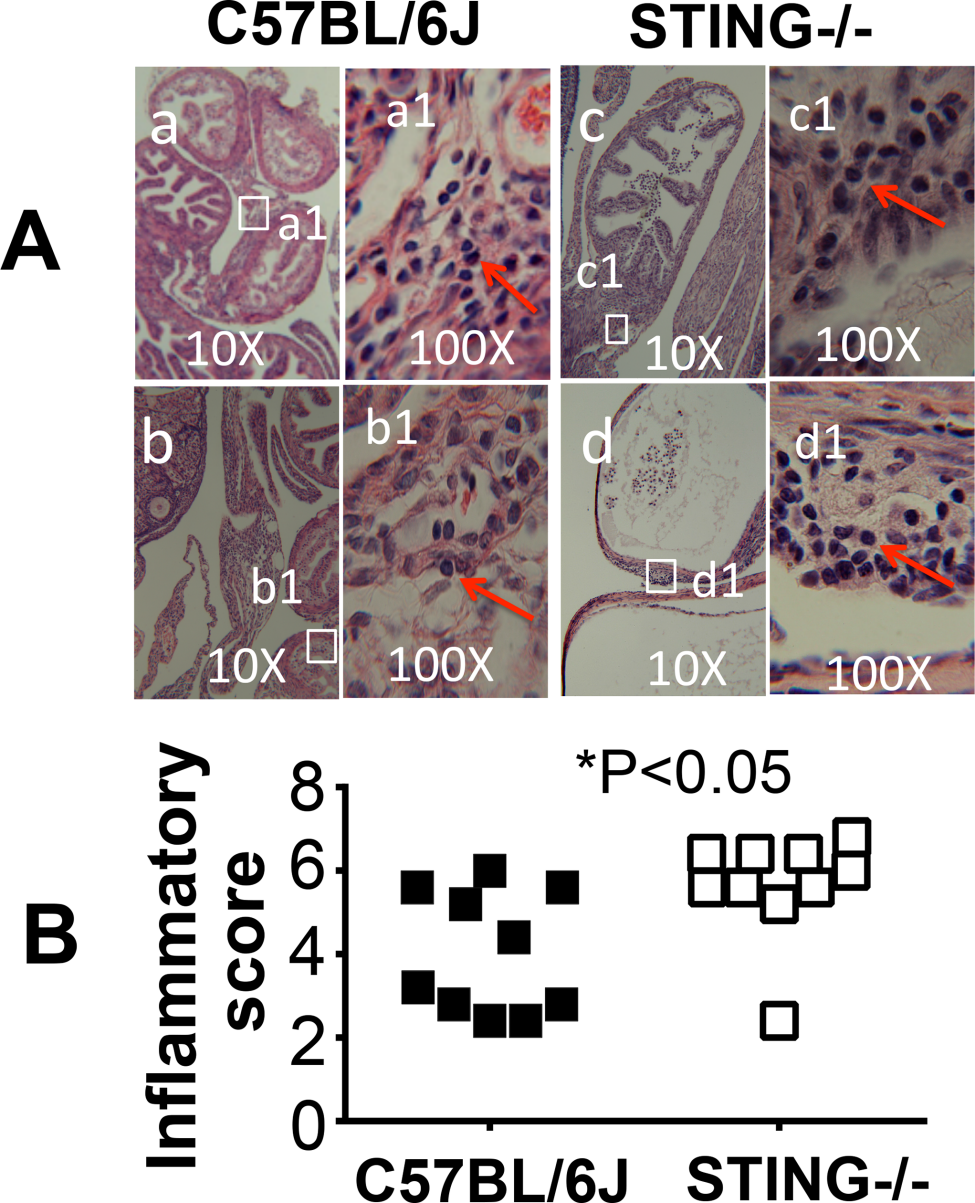
Comparing inflammatory pathology in the oviducts of mice with or without deficiency in STING following a transcervical inoculation. The genital tract tissues from the same mice as described in Fig 7 legend were subjected to H&E staining and evaluation of inflammatory infiltrates in both oviducts. (A) A representative H&E staining image is shown from each group (panels a & b from wild type C57BL/6J mice and c & d from STING-/- mice respectively under 10x objective lens). To visualize the infiltrating cells, examples of a 100x objective lens view are also provided as marked with a1 to d1. The inflammatory cells are marked with red arrows. (B) The H&E stained sections were semi-quantitatively scored based on the scheme described in the materials and method section, and the scores are summarized. Please note that the STING-deficient mice developed more severe inflammatory infiltration in the oviduct tissues (p<0.05, Wilcoxon).

To test whether lack of the uterotubal junction barrier function as a result of the STING pathway deficiency can affect chlamydial pathogenicity in the oviducts, we first compared the infection courses of C57BL/6J and STING-deficient mice following transcervical inoculation with *C*. *muridarum* (Fig 6). The choice of transcervical inoculation was to avoid the interference by the cervical barrier, which has allowed us to focus on the assessment of the uterotubal junction function. We found that mice with or without deficiency in STING developed similar time courses of live organism shedding from the vaginal swabs in terms of both the IFU titers and % of mice positive for IFUs. This suggested that STING deficiency did not affect *C*. *muridarum* colonization in the uterine horn tissues or the lower genital tract tissues following transcervical inoculation. Interestingly, the STING-deficient mice developed significantly higher incidences of bilateral hydrosalpinx (Fig 7). Although 9 out of 10 wild type C57BL/6J mice developed hydrosalpinx, only one mouse developed bilateral hydrosalpinx. However, 8 out of the 10 STING-deficient mice developed bilateral hydrosalpinx (80% vs. 10%, p<0.01, Fisher’s exact test). The hydrosalpinx severity score was also significantly higher in the STING-deficient mice (p<0.01, Wilcoxon). The gross pathology was further confirmed under microscopy (Fig 8), which revealed more significant chronic inflammatory infiltration in the STING-deficient mice.

## Discussion

The *C*. *muridarum* induction of hydrosalpinx mouse model has allowed the identification of both chlamydial [7–11] and host [12–20] factors involved in chlamydial pathogenesis. However, it remains unknown whether the uterotubal junction plays any significant roles in restricting chlamydial ascension. The observation that *E*. *coli* failed to spread freely crossing the uterotubal junction [25] suggests that the uterotubal junction may function as a host defense mechanism for preventing microbial ascension. In the current study, we have provided the first experimental evidence demonstrating a significant role of uterotubal junction in limiting spreading of the sexually transmitted *Chlamydia* infection. First, intrabursally inoculated *C*. *muridarum* failed to efficiently descend to the uterine horn while endometrial *C*. *muridarum* delivered via intrauterine horn inoculation was unable to efficiently ascend to the oviduct. However, the live organisms recovered from different segments of the uterine horn tissue maintained similar titers. These observations have together demonstrated that the uterotubal junction may function as a barrier for preventing *C*. *muridarum* spreading. Second, the uterotubal junction barrier effect was dependent on innate immunity signaling since mice deficient in either MyD88 or STING were no longer able to restrict *C*. *muridarum* spreading crossing the uterotubal junction, which both validated the host defense function of the uterotubal junction and revealed the underlying mechanisms. Finally, mice deficient in STING developed significantly higher incidence of bilateral hydrosalpinx, suggesting that STING-mediated host defense at the uterotubal junction can play a significant role in preventing tubal pathology. However, since the pathology was detected on day 70 after the initial infection and STING is ubiquitously expressed, STING-deficiency in non-uterotubal junction cells may also contribute to the increased pathology. To more precisely define the contribution of the STING pathways in the uterotubal junction to chlamydial infection and pathogenesis, we may need to use conditional knock-out mice, in which STING molecules expressed by uterotubal epithelial cells are selectively deleted.

Given the dominant role of the cervical barrier in preventing microbial ascension by separating the non-sterile lower genital tract from the relatively sterile upper genital tract, the role of the uterotubal junction in restricting microbial spreading to the oviduct was only obvious when the cervical barrier was bypassed. That was why we first used intrabursal versus intrauterine horn inoculations to deliver live *C*. *muridarum* organisms into either side of the uterotubal junction, followed by monitoring the live organism spreading. In this way, the function of the uterotubal junction can be directly measured without any significant interference from the cervical barrier or other mucosal tissues. Using this approach, we have not only demonstrated a significant role of the uterotubal junction in restricting chlamydial spreading but also defined the underlying mechanisms, the latter of which are the innate immunity signaling pathways mediated by MyD88 or STING. To evaluate the relevance of the innate immunity-mediated defense mechanism at the uterotubal junction, we used transcervical inoculation (again allowing us to bypass the cervical barrier) and demonstrated that the STING-dependent uterotubal junction-mediated restriction of chlamydial ascension can be translated into protection against tubal pathology. Our pilot experiments have shown that following an intravaginal inoculation, mice with or without STING developed similar levels of hydrosalpinx (data not shown), suggesting that STING is not important for the function of the cervical barrier. In contrast, we have previously shown that MyD88-deficient mice developed more severe hydrosalpinx following intravaginal infection [14], suggesting an important role of MyD88-dependent pathway in cervical barrier-mediated host defense function in addition to its role in uterotubal junction (see Figs 4 & 5 of the current study). It is clear that the cervical barrier plays a dominant role in preventing chlamydial ascension from the lower genital tract, which over-shadowed the role of the uterotubal junction. Thus, the role of uterotubal junction in restricting chlamydial spreading to the oviduct was only obvious when the cervical barrier was totally bypassed.

Although the overall impact of the uterotubal junction on chlamydial ascension from the lower genital tract is not as significant as that of the cervical barrier, the host defense function of the uterotubal junction may be irreplaceable. The uterotubal junction may represent the last checkpoint for preventing endometrial microbes from reaching the oviduct where a delicate sterile dynamic environment is required for maintaining female reproductive function. Microbial colonization in the endometrial epithelia may be removed periodically during the menstrual or estrus cycle. However, there is no known specialized mechanism for removing tubal colonization by microbes. Thus, prevention of tubal infection via the uterotubal junction becomes extremely important.

The MyD88-mediated signaling pathways have been shown to play significant roles in preventing chlamydial ascending crossing the cervical barrier [14]. In the current study, we have also demonstrated a significant role of the MyD88 pathway in uterotubal junction. Interestingly, deficiency in the cytosolic DNA detection STING pathway also severely compromised the host defense function of the uterotubal junction, suggesting that a functional DNA detection pathway is required for maintaining the normal function of uterotubal junction. It will be interesting to test whether the uterotubal junction-mediated selection for healthy sperm is also dependent on the DNA detection pathway. Although *Chlamydia* has been shown to activate the STING pathway in cell cultures [27, 28], it remained unclear whether *Chlamydia* activates the same pathway during infection in animals and whether the chlamydial activation of STING pathway in animals plays any significant roles in chlamydial pathogenesis. The current study has provided data for addressing these questions. It will be interesting to further map the molecular basis of both host defense and reproductive functions of the uterotubal junction and determine the chlamydial virulence factors that enable *C*. *muridarum* to cross the uterotubal junction. Finally, given the considerable differences in the genital tract anatomy and function between female mice and women, one should be cautious in applying the above knowledge acquired from mouse studies to humans.
